# Light-Controlled Rotational Speed of an Acoustically Levitating Photomobile Polymer Film

**DOI:** 10.3390/ma16020553

**Published:** 2023-01-06

**Authors:** Daniele Eugenio Lucchetta, Paolo Castellini, Milena Martarelli, Lorenzo Scalise, Giuseppe Pandarese, Cristiano Riminesi, Gautam Singh, Andrea Di Donato, Oriano Francescangeli, Riccardo Castagna

**Affiliations:** 1 Dipartimento SIMAU, Università Politecnica delle Marche, Via Brecce Bianche, 60131 Ancona, Italy; 2 Optoacoustic Lab, Dipartimento SIMAU, Università Politecnica delle Marche, Via Brecce Bianche, 60131 Ancona, Italy; 3 Dipartimento DIISM, Università Politecnica delle Marche, Via Brecce Bianche, 60131 Ancona, Italy; 4 CNR, Institute of Heritage Science, Via Madonna del Piano, 10, 50019 Sesto Fiorentino, Italy; 5 Department of Applied Physics, Amity Institute of Applied Sciences, Amity University, Noida 201313, Uttar Pradesh, India; 6 Dipartimento DII, Università Politecnica delle Marche, Via Brecce Bianche, 60131 Ancona, Italy; 7 URT-CNR@UNICAM, Photonic Materials Laboratory, Consiglio Nazionale delle Ricerche (CNR), c/o Università di Camerino (UNICAM), Polo di Chimica, Via Sant’Agostino, 1, 62032 Camerino, Italy

**Keywords:** photomobile film, contactless actuator, acoustic levitation, opto-acoustic motor, acousto-optic rotor

## Abstract

In this work, we study the light-induced changes of the rotational speed of a thin photomobile film using a single-axis acoustic levitator operating at 40 kHz. In our experiments, a 50 μm thick photomobile polymer film (PMP) is placed in one of the nodes of a stationary acoustic field. Under the action of the field, the film remains suspended in air. By externally perturbing this stable equilibrium condition, the film begins to rotate with its natural frequency. The rotations are detected in real time by monitoring the light of a low power He–Ne laser impinging on and reflected by the film itself. During the rotational motion, an external laser source is used to illuminate the PMP film; as a consequence, the film bends and the rotational speed changes by about 20 Hz. This kind of contactless long-distance interaction is an ideal platform for the development and study of many electro-optics devices in microgravity and low-friction conditions. In particular, we believe that this technology could find applications in research fields such as 3D dynamic displays and aerospace applications.

## 1. Introduction

Acoustics levitation is an intriguing emerging field that includes many applications ranging from material sciences and related applications to artistic representations [1,2,3,4,5,6]. Levitation is based on a balance between the weight strength and the generated acoustic radiation force, usually accomplished by one single ultrasonic speaker or by one or more arrays of speakers used in different geometries. The theoretical aspects concerning the stable levitation of axisymmetric Mie objects have recently been reported and analyzed in detail in [7]. Complex 3D movements of particles can be obtained by manipulating the phases of the single speaker/array and using sophisticated combinations of electronics and software. Additionally, systems for assembling objects in a contactless manner using acoustic levitation have recently been reported [5,6,8]. Ultrasonic waves are preferred due to the high amplitude of the acoustic signal necessary to maintain the objects suspended in air. Over the last several years, many different acoustic levitation devices working in one, two, or three dimensions have been proposed. A common arrangement for an acoustic levitator is the single-axis configuration, which can be arranged in two different ways: the first is based on an ultrasonic speaker coupled with a reflector to form a resonant cavity, while the second one is made up of two separate non-resonant emitters. Both configurations have advantages and disadvantages; for example, while resonant devices are very efficient, they are sensitive to changes in temperature and to the spatial configuration of the single elements. While non-resonant devices are not affected by these issues, they require more complex electronics. Both resonant and non-resonant single-axis levitators are driven by a sinusoidal signal to generate a standing wave which traps the particles at its nodes. Moreover, even objects larger than the acoustic wavelength can be successfully and acoustically lifted [9]. With respect to the more well known optical trapping, acoustic trapping has a ratio of trapping force to input energy orders of magnitude higher than optical manipulation. In principle, this allows for the levitation of large and/or heavy objects. Holographic techniques have been proposed to generate complex acoustic fields using only a single ultrasonic source as well as to manipulate multiple objects in an independent way along any spatial direction with speeds approaching 10 m/s [6]. Composite polymer materials, already used in a wide variety of applications such as [10,11,12,13,14,15,16,17,18], play a fundamental role in materials science. Among them, photomobile polymers (PMP), which are materials able to convert light energy into mechanical work, represent the ideal platform for the development of a large variety of touchless devices [19,20,21,22,23,24,25,26,27,28,29,30,31,32,33,34]. In this work, we introduce a novel acousto-optic experimental setup in which small objects rotate around a single axis in space. The device is based on a single-axis levitator containing 72 transducers which are arranged as two surfaces, each containing 36 transducers. In this arrangement, the rotational speed of the objects is controlled by an impinging external light. With this aim, we exploit an object made of PMP-film that we have recently developed and used in different applications. During rotation, the film is illuminated by an external light that changes the conformation of the film itself. The light-induced change in the sample’s shape modifies the inertial moment of the film and, as a consequence, its rotational speed.

## 2. Materials and Methods

### 2.1. Materials

Phenyl-bis(2,4,6-trimethylbenzoyl) phosphine-oxide (I819), 4-aminophenol (4-AP), lead(IV) oxide (PbO_2_), *N*-Vinyl-1-Pyrrolidonone (NVP), and dipentaerythrythol-hydroxy-penta/hexa-acrylate (DPHPA) were from Merck, Darmstadt, Germany.

### 2.2. PMP-Mixture Preparation

We began by oxidating 1 mmol 4-AP in solid form in a small bottle in the presence of 0.25 mmol PbO_2_; the system was left in dark aerobic conditions for one week. After that, NVP (≈5 mmol) was added to the reaction system, which was left in darkness for another seven days in air and at room temperature. Typically, a precipitate formed at the bottom of the bottle, which was carefully removed from the reaction environment. Separately, 1 mmol of DPHPA was blended with 0.14 mmol I819 for 3 h in the dark. Finally, all the components were mixed together in darkness for seven days at room temperature.

### 2.3. PMP-Film Preparation

The PMP-mixture was placed by capillarity in a cell consisting of two glass slides separated by mylar stripes (50 μ thickness). After that, the cell was placed under UV-B irradiation for ≈10 min. Finally, the cell was opened and the PMP-film peeled-off. PMP-films with a mass of 0.001 g having different shapes were used in our experiments.

### 2.4. Optical Setup

The experimental setup used was mainly based on a device called TinyLev, first described in [35]. It consists of a single-axis multi array acoustic levitator operating at 40 kHz. The device produces a stable acoustic field in the central part of the cavity due to the two opposite curved array surfaces, containing 36 transducers each, which provide a well-defined geometric focus. This two-surface system operates in a non-resonant condition and does not require a specific calibration procedure in a wide range of temperatures. The PMP-film is placed in one of the nodes of the acoustic field and rotation is induced by slightly altering the film shape using an external light source or by altering the sound field using laboratory tweezers. A coherent CW He–Ne laser source operating at λ = 633 nm with a power P = 5 mW was used as probe to illuminate the film. The beam reflected by the rotating PMP-film was detected by a photo-diode connected to an oscilloscope. Operating in this way allows the rotational speed of the film to be easily measured. During rotation, other CW laser sources were used to induce changes in the shape of the PMP film. We used a standard DPSS green laser operating at λ = 532 nm and a Coherent Genesis MX SLM blue laser, (Coherent Inc., Santa Clara CA, USA) operating at λ = 460 nm. The lasers impinged on the sample with different orientations until changes in the shape of the PMP films were observed. We used a power of P = 60 mW for both laser sources. A Basler CCD camera, (Basler AG, Ahrensburg, Germany) was used to record a movie of the PMP film levitating and rotating in the acoustic field. The entire system was placed on a damped optical table. The system was further isolated using a cylindrical transparent plastic sheet, and was operated at room temperature.

## 3. Results and Discussion

**Figure 1:**
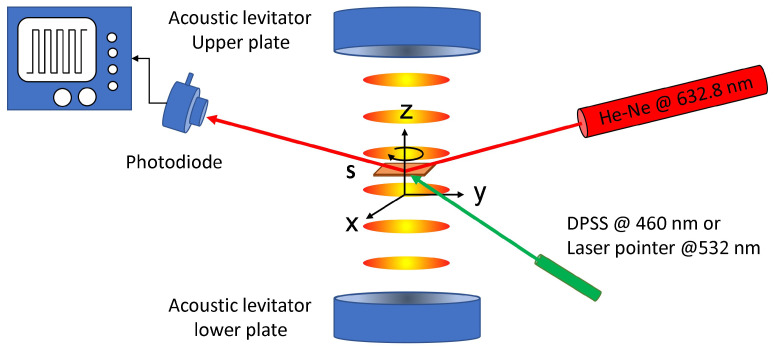
The experimental setup used to measure the changes in the rotational speed of the PMP film. The sample is placed in one of the nodes of the acoustic field. The rotational speed is detected using a photodiode connected to an oscilloscope. The He–Ne laser operating at λ = 632.8 nm is used as probe beam while the DPSS laser is used to induce nonlinear behaviour in the sample.

Acoustic waves at a frequency of 40 kHz have a wavelength of 8.65 mm at 25 °C. This allows the levitation of small objects of dimensions up to λ/2 i.e., ≈4 mm. TinyLev has many advantages: it is made with commercially available and low-cost components, it produces stable trapping, it is not sensitive to small changes in temperature and humidity, it works with low-voltage, it is easy to operate, it does not require calibration, and it can operate for extended periods of time. As a first attempt, we verified the stability of the device using a symmetrical star-shaped object made from a standard plastic material with a thickness of 100 μm and large lateral dimensions. The result is shown in the Supplementary Information Video S1. As can be seen, the star is perfectly stable and does not rotate until an external perturbation is imposed on the system, for example, using tweezers. The theoretical description of the rotation of thin symmetric structures is presently an outstanding object of study. The most convincing opinion states that rotation is probably due to the viscous torque induced by acoustic streaming [36,37,38]. We noticed that asymmetric 2D structures, for example, thin 100 μm thick stars made with asymmetric blades (such as the ones reported in [28]) immediately started to rotate when placed in one node of the acoustic field. PMP with an irregular shape was not an exception. We noticed that irregularly shaped films tended to rotate more easily than symmetrical ones. Symmetrical 2D structures were much more stable until an external perturbation was induced, either mechanically or, as shown here, optically. Figure 1 shows the experimental setup used to perform our rotational speed measurements.

**Figure 2:**
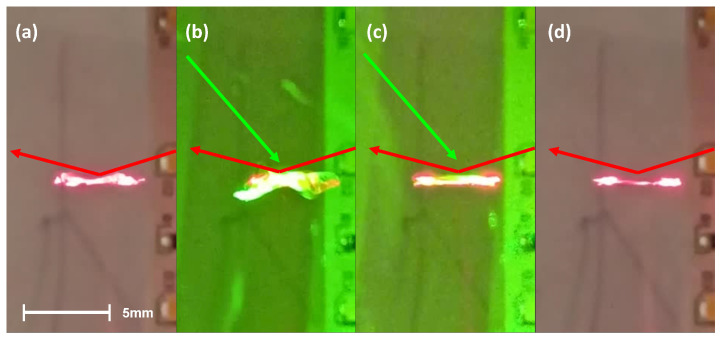
A sequence showing four different states of an irregular shaped PMP film during its rotational motion. The red and green arrows show the probe and pumping laser beams, respectively. In (**a**), the sample rotates in an irregular way; in (**b**), the external perturbation hits the sample, inducing a transient irregular motion; in (**c**), the motion is stabilized; and in (**d**), the sample remains in the more stable conformation for a time before returning to the same state as in (**a**).

The sample is an irregularly shaped PMP film placed in a node of the acoustic field. The equilibrium condition occurs when the gravitational force acting on the film $F_{g}$ = 10 μN is counterbalanced by the opposing radiation force along the z direction. During the acoustic field-induced rotations, the light of the He–Ne CW laser reflected by the PMP sample is detected by a photodiode connected to an oscilloscope. A square wave-like pattern appears on the screen, allowing for direct real-time measurement of the rotational frequency. The external pumping source is the blue light emitted by a DPSS laser or the green light emitted by a laser pointer. Both wavelengths are able to induce shape changes in the PMP film, as evidenced in previous works [24,26,29,30,31,32,33,34]. These light-induced changes are responsible for the measured variation in the rotational speed, as detailed below. Specifically, the arrival of the external pumping light on the sample creates a fast temporary instability followed by a stabilization of the rotational motion. These cases are shown in Figure 2b,c respectively. The edges of the PMP film have an irregular form, visible in Figure 2a, which becomes more flat when the sample is illuminated with an external green light. Depending on the initial shape of the PMP film, this stabilization can induce an increase or decrease in the rotational speed. The result depends on the inertial momentum of the film before and after the shape changes take place. The frames shown in Figure 2 are part of Video S2 reported in the Supplementary Information. The video clearly shows the aforementioned behavior, in which the external pumping light stabilizes the rotational motion of the entire PMP film by flattening its surface. However, situations may occur in which the external pumping beam induces a perturbation strong enough to bring the rotating film out of equilibrium until it is ejected by the lateral acoustic forces (see Video S3).

**Figure 3:**
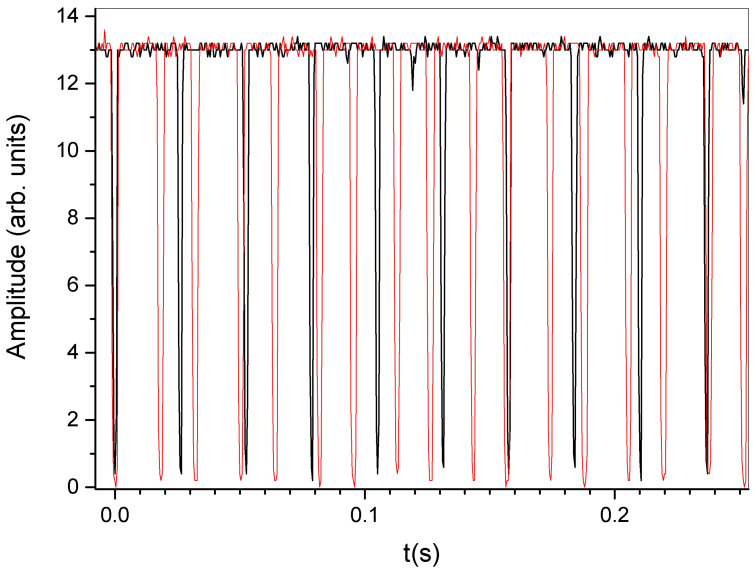
Oscillogram showing the rotational behaviour of the PMP film before (black line) and after (red line) external illumination.

Finally, we performed measurements on a rectangularly shaped PMP film. The film placed in one node is perfectly stable until the end of the film is bent by the external pumping light. The rotation starts with a frequency of ≈38 Hz. By illuminating the whole sample, the PMP film bends in a U-shaped configuration and the frequency appears doubled. The measurements show an increase in the rotational speed of about 20 Hz (from 38 to 55 Hz). Figure 3 clearly shows the changes measured in the rotational speed before and after illumination. In detail, the square wave (the black continuous line) represents the sample status when the external illumination is absent, while the red continuous line represents the final state of the changes induced by the external light irradiation. An increase in the main oscillation period and the appearance of new intermediate peaks due to the light-induced shape deformations can be observed. Video S4 clearly shows the growth in the light-induced deformation and the consequent light-induced changes in the rotational speed.

## 4. Conclusions

In conclusion, in this work we show the rotational behaviour of a PMP film under external illumination. We detected a change in the rotational speed due to light-induced deformations. To the best of our knowledge, this is the first example of a touchless long-distance activable and low friction frequency-tunable motor in microgravity conditions. This technology could find applications in many research fields, including 3D dynamic displays and aerospace applications.

## Data Availability

Data are available from the authors under reasonable request.

## Supplementary Materials

The following supporting information can be downloaded at: https://www.mdpi.com/article/10.3390/ma16020553/s1, Video S1: Externally induced motion on a stable symmetric plastic object; Video S2: light-induced stabilization of the rotational motion of a rotating PMP film; Video S3: light-induced destabilization of the rotational motion of a rotating PMP film; Video S4: Real-time appearance of new intermediate peaks due to the light-induced shape deformations.
